# Prognostic significance of EZH2 expression in patients with oesophageal cancer: a meta‐analysis

**DOI:** 10.1111/jcmm.12791

**Published:** 2016-02-09

**Authors:** Yawei Wang, Fang Gao, Meng Zhao, Bing Li, Dan Xing, Jie Wang, Yang Yang

**Affiliations:** ^1^ Department of ElectromyographyTianjin HospitalTianjinChina; ^2^ Department of GastroenterologyTianjin Dong Li HospitalTianjinChina; ^3^ Department of Laboratory MedicineTianjin Medical University Cancer HospitalTianjinChina; ^4^ Department of OrthopedicsTianjin HospitalTianjinChina

**Keywords:** enhancer of zeste homolog 2, oesophageal cancer, colorectal cancer, gastric cancer, prognosis, meta‐analysis

## Abstract

Enhancer of zeste 2 (EZH2), a key component of polycomb repressive complex 2 (PRC2), was of great importance in human cancer pathogenesis. Various studies examined the relationship between EZH2 overexpression with the clinical outcome in patients with digestive cancers, but yielded conflicting results. Electronic databases updated to January 2015 were searched to find relevant studies. A meta‐analysis was conducted with eligible studies which quantitatively evaluated the relationship between EZH2 overexpression and survival of patients with digestive cancers. Survival data were aggregated and quantitatively analysed. We performed a meta‐analysis of 10 studies (*n* = 1461 patients) that evaluated the correlation between EZH2 overexpression and survival in patients with digestive cancers. Combined hazard ratios suggested that EZH2 overexpression was associated with poor prognosis of overall survival (HR = 1.54, 95% CI: 1.27–1.81) in patients with oesophageal cancer. In the stratified analysis, no significant risks were found among gastric cancer (HR = 0.66, 95% CI: 0.16–1.15) and colorectal cancer (HR = 0.91, 0.63–1.19), indicating that EZH2 was not an indicator of poor prognosis in gastric cancer or colorectal cancer. Enhancer of zeste 2 overexpression indicates a poor prognosis for patients with oesophageal cancer, but not among gastric cancer or colorectal cancer.

## Introduction

Digestive system malignant tumours, with 3.4 million new diagnosed cases and 1.5 million deaths each year, are the most common cancers worldwide 1. Digestive cancers are complex, multistep, multifactorial and highly fatal diseases. Digestive cancers contain alimentary tract and digestive gland cancers. Among them, colorectal, gastric and oesophagus cancers were the common cancers with high incidence and mortality in the world. Despite recent advances in treatment, the prognosis of patients with cancers in digestive system remains poor. Numerous studies have reported molecular predictors of prognosis of patients with digestive system cancers 2, 3, 4. However, no this kind of specific molecular biomarker has been accepted commonly and used routinely until now. The clinically applicable biomarkers for prognosis analysis are urgently required.

Enhancer of zeste homolog 2 (EZH2) is a key component of the polycomb repressive complex 2 (PCR2), which possesses histone methyltransferase activity and mediates gene silencing through posttranslational histone modifications 5. Enhancer of zeste homolog 2 is frequently overexpressed in a wide variety of human malignancies such as breast cancer 6, prostate cancer 7 and lung cancer. In addition, it also promotes cancer development and progression through chromatin modification by epigenetic activation of oncogenic signalling cascades and silencing of tumour suppressor genes, and has been implicated in cell proliferation, differentiation, invasion, and metastasis 8. Thus, it is acting with oncogenic properties.

Many studies have evaluated whether the overexpression of EZH2 may be a prognostic factor for survival in patients with digestive cancers. However, the results of the studies are inconclusive and no consensus has been reached. It is unknown whether differences in these investigations have been mostly because of their limited sample size or genuine heterogeneity. Thus, we conducted a meta‐analysis of all available studies relating EZH2 with the clinical outcome in patients with digestive cancers including oesophageal cancer, gastric cancer and colorectal cancer.

## Materials and methods

### Search strategy and study selection

The electronic databases PubMed, Embase and CNKI (China National Knowledge Infrastructure) were searched for studies to be included in the present meta‐analysis. An upper date limit of January 01, 2015 was applied; we used no lower date limit. Searches included the terms ‘oesophageal or gastric or colorectal’, ‘cancer or carcinoma or tumour or neoplasm’, ‘EZH2’ and ‘prognosis’. We also reviewed the Cochrane Library for relevant articles. The references reported in the identified studies were also used to complete the search.

Studies eligible for inclusion in this meta‐analysis met the following criteria: (*i*) measure EZH2 expression in the primary colorectal cancer or gastric cancer or oesophageal cancer with IHC (immunohistochemistry) or Real Time‐PCR; (*ii*) provide information on survival [*i.e*. overall survival (OS), studies investigating response rates only were excluded]; (*iii*) When the same author reported results obtained from the same patient population in more than one publication, only the most recent report, or the most complete one, was included in the analysis. The authors independently determined study eligibility.

### Data extraction and quality assessment

The final articles included were assessed independently by the reviewers. Data retrieved from the reports included author, publication year, patient source, histology, test method, positive, follow‐up and survival data (Table 1). If data from any of the above categories were not reported in the primary study, items were treated as ‘not applicable’. We did not use pre‐specified quality‐related inclusion or exclusion criteria and did not weigh each study by a quality score, because the quality score has not received general agreement for use in a meta‐analysis, especially experimental observational studies 9.

**Table 1:** Main characteristics and results of the eligible studies

First author‐year	Patients source	Histology	Stage	N pts	Method	Positive (%)	HR estimation	Survival results
Benard 2014	Netherlands	Colorectal cancer	I–IV	408	IHC	NA	HR and 95% CI 0.84 (0.60–1.18)	NS
Liu 2014	China	Colorectal cancer	I–IV	82	Real time‐PCR	NA	HR and 95% CI 2.51 (1.10–5.74)	Poor
Wang 2010	China	Colorectal cancer	I–IV	119	IHC	32.8	HR and 95% CI 3.21 (1.06–9.73)	Poor
Kodach LL 2010	Netherlands	Colorectal cancer	NA	72	IHC	46	Survival curves 1.42 (0.65–2.93)	NS
He 2012	China	Gastric cancer	I–IV	117	IHC	70	HR and 95% CI 1.88 (1.17–3.03)	Poor
Lee 2012	South Korea	Gastric cancer	I–IV	178	IHC	92.1	HR and 95% CI 0.11 (0.01–1.20)	NS
Matsukawa 2006	Japan	Gastric cancer	I–IV	83	IHC	56.6	Survival curves 1.72 (0.06–5.89)	NS
Ha 2008	South Korea	Oesophageal cancer	I–IV	164	IHC	52.4	Survival curves 1.24 (0.83–1.68)	NS
Wang H 2013	China	Oesophageal cancer	I–IV	102	IHC	65.7	Survival curves 2.47 (1.75–3.43)	Poor
Yamada 2011	Japan	Oesophageal cancer	I–IV	136	IHC	14	Survival curves 1.59 (1.25–2.03)	Poor

IHC: immunohistochemistry; NS: not significant; NA: not applicable; HR: hazard ratio; N pts: number of patients; PCR: polymerase chain reaction.

### Statistical methods

For the quantitative aggregation of the survival results, we measured the impact of EZH2 overexpression on survival by HR between the two survival distributions. HRs and 95% confidence intervals (CIs) were used to combine as the effective value. If the HRs and their 95% CIs were given explicitly in the articles, we used crude ones. When these variables were not given explicitly, they were calculated from the available numerical data using methods reported by Parmar *et al*. 10.

Heterogeneity of the individual HRs was calculated with chi‐squared tests according to Peto's method 11. Heterogeneity test with inconsistency index (Ι^2^) statistic and *Q* statistic was performed. If HRs were found to have fine homogeneity, a fixed effect model was used for secondary analysis; if not, a random‐effect model was used. DerSimonian‐Laird random effects analysis 12 was used to estimate the effect of EZH2 overexpression on survival. By convention, an observed HR >1 implies worse survival for the group with EZH2 overexpression. The impact of EZH2 on survival was considered to be statistically significant if the 95% CI did not overlap with 1. Horizontal lines represent 95% CIs. Each box represents the HR point estimate, and its area is proportional to the weight of the study. The diamond (and broken line) represents the overall summary estimate, with CI represented by its width. The unbroken vertical line is set at the null value (HR = 1.0).

Evidence of publication bias was sought using the methods of Egger *et al*. 13 and of Begg and Mazumdar 14. Intercept significance was determined by the *t*‐test suggested by Egger (*P* < 0.05 was considered representative of statistically significant publication bias). All of the calculations were performed by STATA version 11.0 (Stata Corporation, College Station, TX, USA).

## Results

### Study selection and characteristics

Ten studies 15, 16, 17, 18, 19, 20, 21, 22, 23, 24 published between 2006 and 2014 were eligible for this meta‐analysis. All reported the prognostic value of EZH2 status for survival in colorectal cancer or gastric cancer or oesophageal cancer patients. The total number of patients included was 1461, ranging from 82 to 408 patients per study (median 146). The major characteristics of the 10 eligible publications are reported in Table 1. The studies were conducted in 4 countries (China, South Korea, The Netherlands and Japan). Among the 10 studies, eight studies (981 patients, 67.1%) were performed in Asian populations, and the remaining two studies (480 patients, 32.9%) followed Netherlands patients. All patients in the eligible studies were determined by pathological stage.

All of the studies reported the prognostic value of EZH2 status for survival in patients with lung cancer. Of the 10 studies, five directly reported HRs (multivariate analysis), while the other five studies provided survival curves. Five of the 10 studies identified EZH2 overexpression as an indicator of poor OS, and the other five studies showed no statistically significant impact of EZH2 overexpression on OS.

### Meta‐analysis

**Figure 1:**
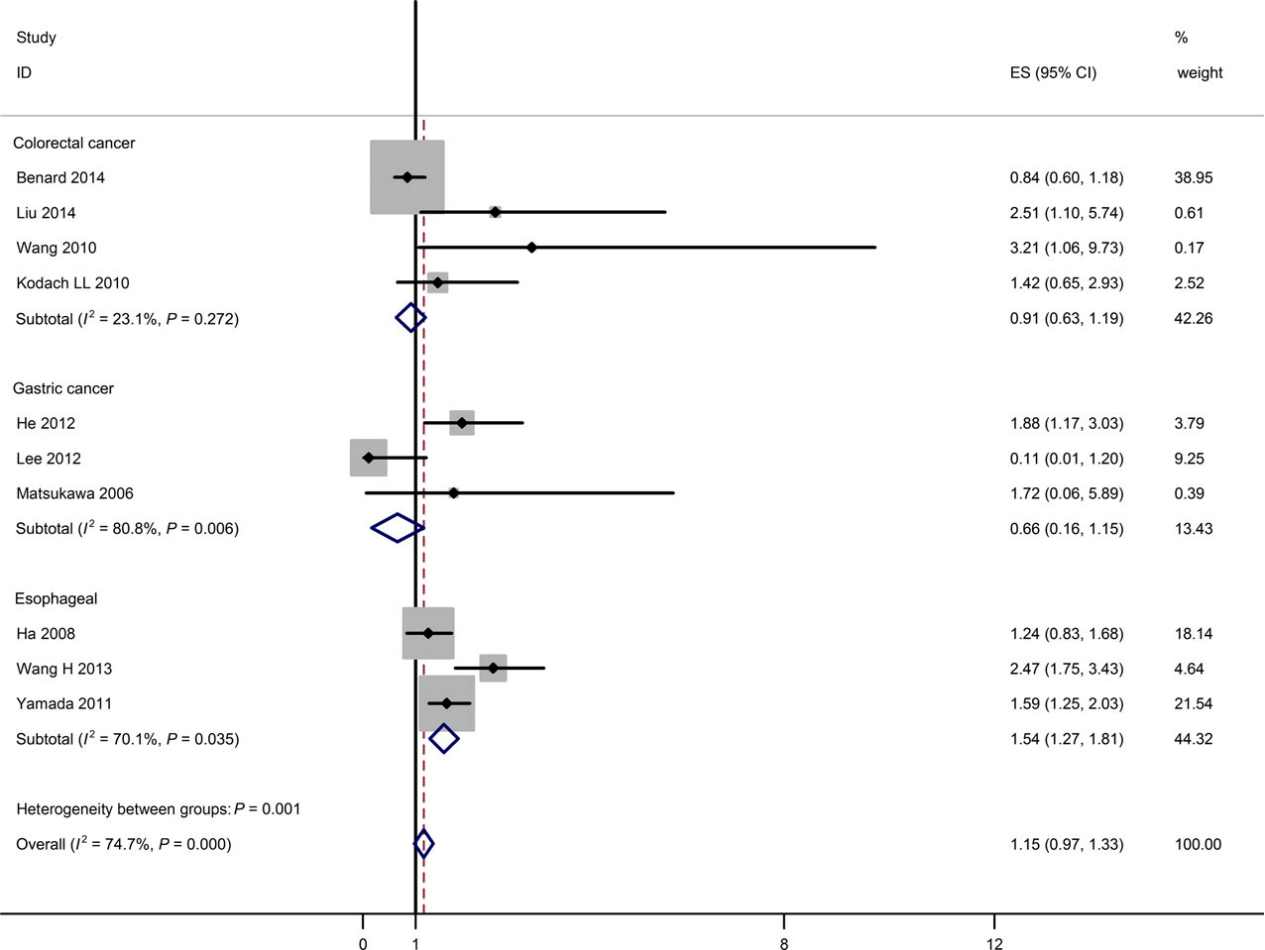
Meta‐analysis (Forest plot) of the 10 evaluable studies assessing EZH2 in patients with digestive cancers stratified by different histological types for overall survival.

The results of the meta‐analysis were shown in Table 2 and Figure 1. Overall, the combined HR for all 10 eligible studies evaluating EZH2 overexpression on OS was (HR = 1.15, 95% CI: 0.97–1.33), suggesting that EZH2 overexpression was not associated with poor prognosis for combined effect of the three digestive cancer. No significant heterogeneity was observed among the studies (*Q* = 5.89, *I* ^2^ = 74.7%, *P* = 0.000).

**Table 2:** Meta‐analysis: HR value in colorectal cancer, gastric cancer and oesophageal cancer

	Nb	Patients	Combined HR (95% CI)	Chi‐squared heterogeneity test (*P*)
Overall	10	1461	1.15 (0.97–1.33)	0.000
Esophageal cancer	3	402	1.54 (1.27–1.81)	0.035
Gastric cancer	3	378	0.66 (0.16–1.15)	0.006
Colorectal cancer	4	681	0.91 (0.63–1.19)	0.272

HR: hazard ratio; Nb: number of studies; CIs: confidence intervals.

When grouped according to the various histological types of digestive cancer, the combined HRs of oesophageal cancer was (HR = 1.54, 95% CI: 1.27–1.81), indicating EZH2 was an indicator of poor prognosis in oesophageal cancer (*P* = 0.035 for heterogeneity test). However, the combined HRs of gastric cancer and colorectal cancer were (HR = 0.66, 95% CI: 0.16–1.15) and (HR = 0.91, 95% CI: 0.63–1.19), respectively, indicating EZH2 was not an indicator of poor prognosis in gastric cancer or colorectal cancer.

### Publication bias

**Figure 2:**
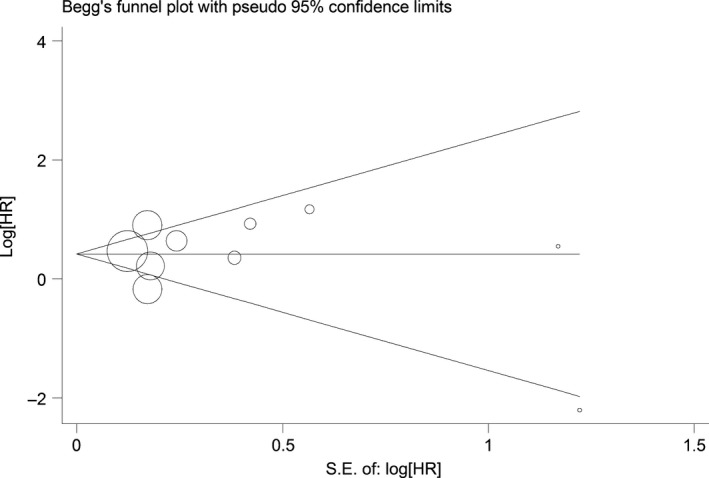
Funnel plot of the 10 evaluable studies assessing EZH2 in patients with digestive cancers for overall survival.

Begg's funnel plot and Egger's test were performed to assess the publication bias in the literature. All 10 eligible studies investigating EZH2 overexpression on OS yielded a Begg's test score of *P* = 0.348 and an Egger's test score of *P* = 0.461, meanwhile according to the funnel plot (Fig. 2), the absence of publication bias was found. Similar results were found for investigating EZH2 overexpression on OS of the three digestive cancers. These results suggested that there were no publication biases in the subgroup analyses.

## Discussion

Enhancer of zeste homolog 2 is the catalytic subunit of the PRC2 protein complex. Trimethylation of lysine 27 on histone H3 (H3K27me3), a marker of transcriptionally silent chromatin, is methylated by EZH2 25, 26. The gene of EZH2, plays an important role in tumorigenesis and cancer progression through epigenetic gene silencing and chromatin remodelling 7. Enhancer of zeste homolog 2 is also capable of keeping the transcriptional repressive state of genes over successive cell generations 26. Disruption of EZH2 expression restricts cell proliferation and induces cell cycle arrest at the G2 phase, whereas the overexpression of EZH2 can shorten the G1 phase of the cell cycle and lead to cell accumulation in the S phase 27. Furthermore, EZH2 protein, as a transcriptional repressor, may help the induction of transcriptional repression and participation in the controlling of gene expression patterns in the gastric epithelial cells, thereby resulting in the loss of tumour suppressor functions 28.

Our present meta‐analysis is the first to evaluate the correlation between EZH2 overexpression and survival in patients with digestive cancers. This meta‐analysis combined 10 publications including 1461 patients with lung cancer to yield statistics, indicating different roles of EZH2 on OS in oesophageal cancer, gastric cancer and colorectal cancer. Combined hazard ratios suggested that EZH2 overexpression was associated with poor prognosis of OS (HR = 1.54, 95% CI: 1.27–1.81) in patients with oesophageal cancer. In the stratified analysis by histological types, significantly risks were not found among gastric cancer (HR = 0.66, 95% CI: 0.16–1.15) or colorectal cancer (HR = 0.91, 95% CI: 0.63–1.19), respectively, indicating EZH2 was not an indicator of poor prognosis in gastric cancer or colorectal cancer.

The heterogeneity issue was complicated in the systematic review and meta‐analysis was. We found no significant heterogeneity among all studies included and subgroup analysis. Another potential source of bias is related to the method of HR and 95% CI extrapolation. If these statistics were not reported by the authors, we calculated them from the data available in the article. If this was not possible, we extrapolated them from the survival curves, necessarily making assumptions about the censoring process. Data for multivariate survival analysis reported in the article were included in the present systematic review with meta‐analysis; if these data were not available, data calculated from survival curves by univariate analysis were included. These results should be confirmed by an adequately designed prospective study. Furthermore, the exact value of EZH2 overexpression status needs to be determined by appropriate multivariate analysis. Unfortunately, few prospectively designed prognostic studies concerning biomarkers have been reported; thus, our collection of many retrospective studies revealed more significance.

Publication bias 29 is a major concern for all forms of meta‐analysis; positive results tend to be accepted by journals, while negative results are often rejected or not even submitted. The present analysis does not support publication bias; the obtained summary statistics likely approximate the actual average. However, it should be noted that our meta‐analysis could not completely exclude biases. For example, the study was restricted to papers published in English and Chinese, which probably introduced bias.

To sum up, our meta‐analysis is the first study to systematically estimate the association between EZH2 expression detected by IHC or Real time‐PCR and survival of patients with digestive cancers. As determined in our meta‐analysis, we concluded that EZH2 overexpression was associated with poor OS in oesophageal cancer, but not among gastric cancer or colorectal cancer. Thus, the detection of EZH2 expression may be of great value in determining the prognosis of oesophageal cancer patients. However, given the limitations of our meta‐analysis, further studies with more integral data and larger sample sizes are required to achieve a more widely applicable statistical analysis.

## Conflicts of interest

The authors declare no any conflicts of interest in this work.
